# Breast Cancer Classification Depends on the Dynamic Dipper Throated Optimization Algorithm

**DOI:** 10.3390/biomimetics8020163

**Published:** 2023-04-17

**Authors:** Amel Ali Alhussan, Marwa M. Eid, S. K. Towfek, Doaa Sami Khafaga

**Affiliations:** 1 Department of Computer Sciences, College of Computer and Information Sciences, Princess Nourah bint Abdulrahman University, P.O. Box 84428, Riyadh 11671, Saudi Arabia; 2 Faculty of Artificial Intelligence, Delta University for Science and Technology, Mansoura 35712, Egypt; 3 Delta Higher Institute for Engineering and Technology, Mansoura 35111, Egypt; 4 Computer Science and Intelligent Systems Research Center, Blacksburg, VA 24060, USA

**Keywords:** breast cancer, deep learning, feature selection, dipper throated optimization algorithm, particle swarm optimization algorithm

## Abstract

According to the American Cancer Society, breast cancer is the second largest cause of mortality among women after lung cancer. Women’s death rates can be decreased if breast cancer is diagnosed and treated early. Due to the lengthy duration of manual breast cancer diagnosis, an automated approach is necessary for early cancer identification. This research proposes a novel framework integrating metaheuristic optimization with deep learning and feature selection for robustly classifying breast cancer from ultrasound images. The structure of the proposed methodology consists of five stages, namely, data augmentation to improve the learning of convolutional neural network (CNN) models, transfer learning using GoogleNet deep network for feature extraction, selection of the best set of features using a novel optimization algorithm based on a hybrid of dipper throated and particle swarm optimization algorithms, and classification of the selected features using CNN optimized using the proposed optimization algorithm. To prove the effectiveness of the proposed approach, a set of experiments were conducted on a breast cancer dataset, freely available on Kaggle, to evaluate the performance of the proposed feature selection method and the performance of the optimized CNN. In addition, statistical tests were established to study the stability and difference of the proposed approach compared to state-of-the-art approaches. The achieved results confirmed the superiority of the proposed approach with a classification accuracy of 98.1%, which is better than the other approaches considered in the conducted experiments.

## 1. Introduction

One of the most frequent types of cancer in women, breast cancer, develops in the breast and then spreads to other regions of the body [1]. Breast cancer is the second most frequent malignancy worldwide (after lung cancer) [2,3]. In the case of breast cancer, the cancer that forms can sometimes be detected using X-ray. Approximately 1.8 million new instances of cancer will be identified in 2020 [4]. Of these, breast cancer will account for almost 30%. Various properties of cells are used to organize them into distinct categories. Malignant breast cancer is one kind, while benign breast cancer is another. Therefore, if the death rate associated with breast cancer is required to be lowered, then early detection is essential [5].

Several imaging methods exist for diagnosing breast cancer at an early stage. In clinical practice, breast ultrasonography is one of the most often utilized diagnostic tools [6,7]. Breast cancer originates in the epithelial cells that line the lobular unit of the terminal duct. Cancer cells that do not invade other tissues are called noninvasive [8]. In in situ cancer, cells are inside the basement membranes of the draining duct and the terminal duct lobular unit. The presence or absence of metastases to the axillary lymph nodes is a major determinant in determining the best course of therapy for breast cancer [9]. When diagnosing and classifying breast problems, ultrasound imaging is among the most popular diagnostic tools [10]. When it comes to cancer, radiological diagnosis is one of the most popular imaging modalities employed, alongside mammography. Nothing is ever said about the potential difficulties it could face in the actual world. There must be careful consideration given to the existence of speckle, and preprocessing techniques such as wavelet-based denoising [11] should be considered in the first and second generations [12].

Ultrasound is a powerful diagnostic technique in dense breast tissue, often detecting breast cancers that mammography misses [13]. Ultrasound is widely utilized in diagnosing breast cancers [9] because it is non-invasive, generally well tolerated by women, and does not expose patients to radiation. Ultrasound imaging is more portable and less expensive than other medical imaging modalities such as MRI and mammography [14]. To aid radiologists in evaluating breast ultrasound testing, computer-aided diagnosis (CAD) systems were created [15,16]. It is challenging to generalize the visual information used by older CAD systems [17,18,19,20,21,22] to ultrasound images captured using various techniques. AI methods for automated breast cancer detection utilizing ultrasound images have made great strides forward in recent years [23,24,25]. Key components of an automated procedure include ultrasound image preprocessing, cancer segmentation, feature extraction from segmented cancer, and classification [26].

Deep learning has recently demonstrated significant improvement in several areas, including cell segmentation [27], skin melanoma identification [28], hemorrhage detection [29], and a few other areas [30,31]. Clinical applications of deep learning in medical imaging have been fruitful, particularly in the detection of breast cancer [32], COVID-19 [33], Alzheimer’s disease identification [34], and brain cancer [35] diagnoses, among others [36,37,38]. The convolutional neural network (CNN) is a multi-layered deep learning architecture. A CNN can take image data and turn it into usable features. Infection detection and categorization are two applications that make use of the attributes. CNN uses feature extraction to retrieve information from the original images. The feature extraction process also generates some irrelevant features from the raw images, which might have a negative impact on classification accuracy. Therefore, for a higher classification accuracy rate, it is crucial to choose the most significant features [39]. Research on how to pick the most useful features from a set of extracted features is ongoing. Selection techniques such as the Genetic Algorithm (GA), Particle Swarm Optimization (PSO), and others have been introduced in the literature and used in medical imaging. These strategies allow us to focus on the optimal subset of features rather than the full feature space. Methods for selecting relevant features have the dual benefit of increasing system accuracy and minimizing processing time [40]. However, a few crucial properties are sometimes overlooked during optimal feature selection, impacting system accuracy. Thus, scientists working in computer vision have developed methods of fusing different types of information together [41]. The fusion procedure boosts the system’s efficacy by multiplying the number of predictors [42]. Serial and parallel fusion are two common feature fusion methods [43].

Within the scope of this paper, the following issues are examined: (i) As a model based on a smaller number of images produces inaccurate prediction, the available ultrasound data are insufficient for building a good deep model. (ii) Misclassification often occurs because of the great degree of resemblance between benign and malignant breast cancer cancers. (iii) Incorrect predictions are made because the features retrieved from images include redundant and unimportant data. To address these issues, a novel approach is proposed based on deep-learning-based and metaheuristic optimization for breast cancer classification from ultrasound images.

The remaining sections of this article are presented as follows. Section 2 presents a discussion of the work that inspired this article. Specifically, deep learning, feature selection, and fusion are presented in Section 3. In Section 4, the results are analyzed and debated. In Section 5, the proposed methodology is wrapped up.

## 2. Literature Review

Researchers have presented several automated approaches for breast cancer categorization using ultrasound images [44,45]. These methods are based on computer vision. A subset of these researchers collected features from raw images, whereas others focused on the segmentation phase first [46]. In a few instances, researchers employed the preprocessing procedure to increase contrast in the input images and to emphasize the infected region for more accurate feature extraction [47]. One such CAD approach was described for breast cancer screening in [48]. Hilbert Transform (HT) was used to convert the raw data into a brightness-mode image to create the final product. The cancer was then divided into smaller regions using a watershed transformation that was governed by markers. After that, the ensemble decision tree model and the K-nearest neighbor (KNN) classifier were used to extract form and textural data and to classify them. The authors in [3] used semantic segmentation, fuzzy logic, and deep learning to segment and classify breast cancers from ultrasound images. The cancer was segmented using a semantic segmentation strategy after being preprocessed using fuzzy logic. Tumors were eventually classified using one of eight previously trained models.

The radionics classification pipeline was first presented by the authors in [49] and is based on machine learning (ML). This was accomplished by isolating the ROI and extracting the interest features. Machine learning classifiers were used to assign categories to the retrieved features. Results from experiments performed on the breast ultrasound images (BUSI) dataset indicated a rise in precision. The authors in [14] presented a deep learning-based system to classify breast mass from ultrasound images. Improved data transmission was achieved via transfer learning (TL) and deep representation scaling (DRS) layers inserted between blocks of pre-trained CNN. Only the parameters of the DRS layers were altered during network training to adapt the previously trained CNN to assess breast mass categorization from the input images. The DRS approach performed exceptionally well in comparison to state-of-the-art methods, as evidenced by the findings. The authors in [5] presented a Dilated Semantic Segmentation Network (Di-CNN) to find and label breast cancer. When extracting features, they looked to a pre-trained DenseNet201 deep model and refined it through transfer learning. Further, the nodules were categorized using a pre-trained model and a 24-layer CNN that fused feature information in parallel. The findings demonstrated that the fusion procedure enhanced the recognition accuracy.

A contextual level set technique was described for breast cancer segmentation in [50]. A network based on UNet-like encoder-decoder architecture was developed to acquire broad semantic context. The breast cancer classification network proposed by the authors in [51] is a deep, doubly supervised transfer learning model. Specifically, they implemented the Maximum Mean Discrepancy (MMD) criteria within a learning context using the Privileged Information (LUPI) paradigm. Later, they integrated the methods into a new doubly supervised TL network (DDSTN), outperforming both methods individually. In [52], Woo et al. presented an automated technique for analyzing ultrasound images to classify breast cancer. Using numerous convolutional neural network (CNN) models, they coupled them with a new image fusion approach. This approach was experimentalized on both public and private (private) datasets, with impressive results. The authors in [53] introduced a deep learning model to detect breast masses in ultrasound images. Different sizes, shapes, and phenotypes of breast masses were considered. The team used selective kernel U-Net CNN to address these concerns. Using this method, they combined the data and utilized them to experiment on 882 breast images. Moreover, they took into account three additional datasets and enhanced their accuracy.

Breast MRI slices can be analyzed using a computerized method developed by the authors in [54] to find breast tumor sections (BTS). The BTS is enhanced and extracted from 2D MRI slices using a combination thresholding and segmentation strategy. To enhance the BTS, the authors develop tri-level thresholding using the Slime Mould Algorithm and Shannon’s entropy. Then, the authors use watershed segmentation to mine the BTS for useful information. After the BTS were extracted, they were compared to the ground truth to obtain the necessary image performance values. Breast cancer was detected with the use of an extreme learning machine (ELM) by the authors in [55]. Second, irrelevant features were filtered out by employing the gain ratio feature selection strategy. At last, the authors demonstrated and validated a cloud-based approach to remote breast cancer diagnoses on the Wisconsin Diagnostic Breast Cancer dataset.

With edge detection and the U-NET model, the authors in [56] proposed a method for diagnosing brain tumors. Image enhancement, edge detection, and classification utilizing fuzzy logic form the basis of the proposed tumor segmentation system by preprocessing the input images with a contrast enhancement strategy, then using a fuzzy logic-based edge detection method to locate the edge in the original images, and finally using a dual tree-complex wavelet transform at various scale levels. To identify meningioma in brain scans, the authors used the fading sub-band images to calculate the features, which were subsequently categorized using the U-NET CNN classification. Breast thermal images were used to develop an automated breast cancer diagnostic system by the authors in [57]. In the first step, they photographed women with their breasts in a variety of positions. Extensive connection and sophisticated coarse segmentation were used in this research. In subsequent steps, the nodule was honed using a dilated filter. Additionally, the accuracy of the proposed architecture was recommended to be enhanced by introducing a class imbalance loss function.

According to the methods discussed, researchers often overlook the preprocessing stage. The researchers often began with the segmentation process and then moved on to the feature extraction phase. To enhance their categorization accuracy, several of them tried feature fusion. Unfortunately, they did not put much effort into picking the best features. Computational time is now a significant consideration they did not consider. In this study, we put forth a best practice for classifying breast masses using a combination of several deep-learning features. Table 1 summarizes some of the most cutting-edge methods.

## 3. The Proposed Methodology

**Figure 1:**
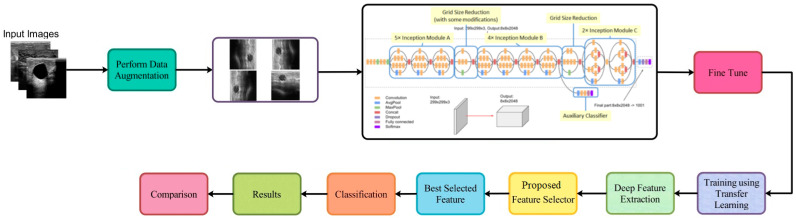
Architecture of the proposed methodology.

This section describes the proposed framework for ultrasonic breast imaging breast cancer categorization. For a visual representation of the proposed framework’s structure, see Figure 1. The original ultrasound images are first enhanced with additional data before being sent into GoogleNet, a fine-tuned deep network, to be trained. To train, transfer learning is utilized to draw features from a global average pool layer. Improved optimization methods for the extracted features, such as the dipper throated and particle swarm varieties, are used. By applying a probabilistic method, the most useful features are combined. A machine learning classifier is then utilized to categorize the fused features.

### 3.1. Dataset Augmentation

**Figure 2:**
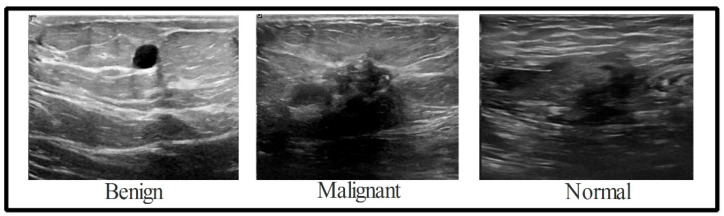
The three classes of the samples in the adopted dataset.

Recent years have seen significant research on data augmentation’s role in deep learning. However, available datasets in the medical sector are considered to be a low resource, which is a problem for deep learning, as it requires a large number of training samples. Because of this, a data augmentation process is required to boost the variety of the primary dataset. When validating these results, the BUSI dataset is consulted. There are 780 images (500 × 500) in the dataset, with 133 “normal” images, 210 “malignant” images, and 487 “benign” images [58], as shown in Figure 2. All of these data were split in half for use in training and testing. Following this, the normal (56 images), malignant (105 images), and benign images used for training were all collected (243 images). The deep learning model cannot be trained without an additional data augmentation phase. The original ultrasound images are implemented and processed by three different operations, including horizontal flip, vertical flip, and rotation 90 degrees, to expand the variety of the original dataset. The implemented actions are repeated until there are 4000 images in each class. With the enhancements applied, the dataset now contains 12,000 images.

### 3.2. Transfer Learning

**Figure 3:**
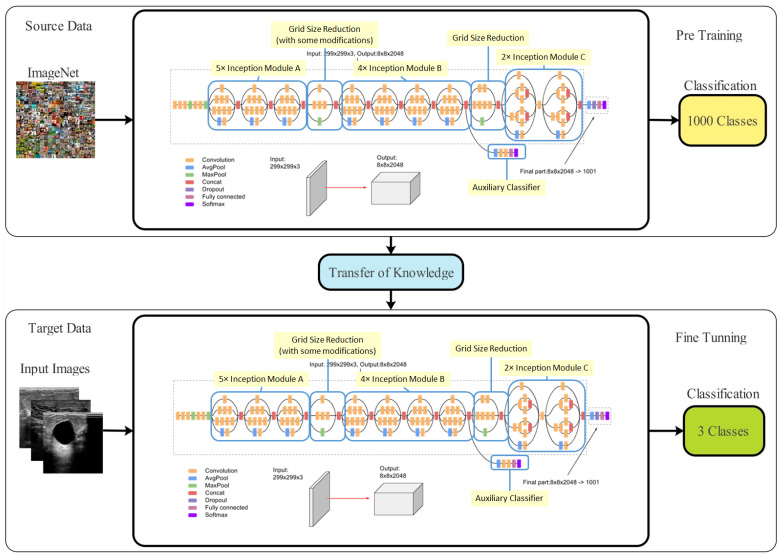
Architecture of the transfer learning process.

Transfer learning (TL) is a machine learning technique that applies a previously learned model to a new problem. From a practical aspect, a supervised learning agent’s sampling efficiency may be vastly improved by reusing or transferring data from previously taught tasks for the newly learned tasks [59]. Deep feature extraction is performed using TL in this case. To do this, first a model that has been pre-trained is adjusted, and then, another model is trained using TL. An updated deep model is trained using information from the previous model (source domain) (target domain). The new model is then trained with the following hyperparameters: learning rate = 0.001, mini-batch size = 16, epochs = 200, and learning technique = stochastic gradient descent. These features are derived from the modified deep model’s Global Average Pooling (GAP) layer. Then, two reformed optimization techniques are used to fine-tune the retrieved features. Figure 3 depicts a graphical representation of the transfer learning process.

### 3.3. Classification of Selected Features

The proposed feature selection method selects the most significant features that can be used to robustly categorize the breast cancer case. The classification of the selected features is performed in terms of a convolutional neural network (CNN). The performance of this CNN is boosted by utilizing the proposed optimization algorithm for optimizing the parameters of the CNN. The optimized CNN is then used to classify the selected features. The output of the optimized CNN is analyzed and evaluated using several criteria and is finally compared to the recent classification models to demonstrate its effectiveness and superiority.

### 3.4. The Proposed Optimization Algorithm

**Figure 4:**
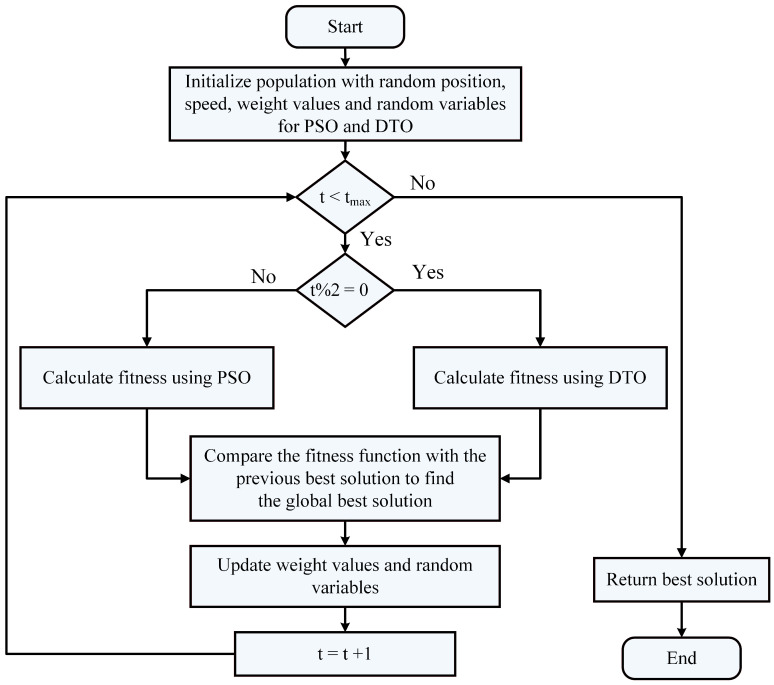
Flowchart of the proposed DDTPSO algorithm.

The proposed optimization algorithm is based on two optimization algorithms, namely, dipper throated optimization (DTO) and particle swarm optimization (PSO) algorithms, which swaps between the two algorithms dynamically and is denoted by dynamic DTPSO (DDPSO). The proposed algorithm exploits the advantages of both algorithms to improve the exploration and exploitation of the optimization process and thus finds the best solution optimally. The steps of the proposed algorithm are shown in the flowchart depicted in Figure 4.

### 3.5. Feature Selection Algorithm

The feature selection process proposed in this work is based on the proposed optimization algorithm but with converting the resulting solution to binary. Therefore, the proposed feature selection method is denoted by binary DDTPSO (bDDTPSO). The conversion to binary is based on the sigmoid function that converts the result of the continuous DDTPSO into binary. The proposed feature selection algorithm is presented in Algorithm 1. This algorithm is used to select the significant features resulting from the feature extraction process performed using the GoogleNet deep network. The selected features are then used to classify the input ultrasound image of a breast to determine the case of cancer.    

**Algorithm 1:** The proposed binary DDTPSO algorithm.1: **Initialize** the parameters of DDPSO algorithm2: **Convert** the resulting best solution to binary [0, 1]3: **Evaluate** the fitness of the resulting solutions4: **Train** KNN to assess the resulting solutions5: **Set**
*t* = 16: **while**
$\;t\leq Max_{iteration}\;$
**do**7:  **Run** DDPSO algorithm to obtain best solutions $S_{best}$8:  **Convert** best solutions to binary using the following equation:              $\begin{array}{c} S_{binary}=\left\{\begin{array}{cc} 1 & \mathrm{if}\;F(S_{best})\geq 0.50\\ 0 & otherwise \end{array}\right.,\; \end{array}$

             $F\left(S_{Best}\right)=\frac{1}{1+e^{-10(S_{Best}-0.5)}}$9:    **Calculate** the fitness value10:  **Update** the parameters of DDTPSO algorithm11:  **Update**
*t* = *t* + 112: **end while**13: **Return** best set of features

## 4. Experimental Results

The specifications of the machine used to run the conducted experiment are: Windows 11 PC with Core(TM) i5-2430M CPU @ 2.40 GHz and 16 GB of RAM and NVidia GPU with 8 GB memory. Python 3.10 is also used to implement the proposed methodology. A freely available dataset on Kaggle is used to train the proposed methodology for classifying breast cancer cases. The dataset’s training, validation, and testing subsets are all assigned identical random sizes. The testing set is used to evaluate the effectiveness of the provided model, whereas validation is employed while calculating the fitness function for a given solution.

### 4.1. Configuration Parameters

The experimental setup for the proposed approach is shown in Table 2 for the optimization methods employed in this work. Ten search agents are used for each optimization algorithm with 80 iterations and 20 runs. A k-fold cross-validation value of 10 is applied while training the classification models.

### 4.2. Evaluation Metrics

The achieved results are assessed using the criteria presented in Table 3. The criteria listed in this table are used to evaluate the performance of the proposed feature selection method [60,61,62,63,64,65]. In addition, the criteria listed in this table are used to assess the performance of the proposed optimized classification model. In this table, the number of runs is denoted by *M*, the best solution at run *j* is denoted by $S_{j}^{*}$, and $size(S_{j}^{*})$ refers to the best solution vector length. In addition, the size of the test set is denoted by *N*, and the predicted and actual values are denoted by $\hat{V_{n}}$ and $V_{n}$, respectively. Moreover, the true positive, true negative, false positive, and false negative are denoted by TP, TN, FP, and FN, respectively.

**Table 2:** Configuration parameters of the optimization algorithms.

Algorithm	Parameter	Value
DTO [66]	Iterations	500
	Number of runs	30
	Exploration percentage	70
PSO [67]	Acceleration constants	[2, 12]
	Inertia $W_{max}$, $W_{min}$	[0.6, 0.9]
	Number of particles	10
	Number of iterations	80
WOA [68]	*r*	[0, 1]
	Number of iterations	80
	Number of whales	10
	*a*	2 to 0
GWO [69]	*a*	2 to 0
	Number of iterations	80
	Number of wolves	10
SBO [70]	Step size	0.94
	Mutation probability	0.05
	Lowe and upper limit difference	0.02
GA [71]	Cross over	0.9
	Mutation ratio	0.1
	Selection mechanism	Roulette wheel
	Number of iterations	80
	Number of agents	10
MVO [72]	Wormhole existence probability	[0.2, 1]
FA [73]	Number of fireflies	10
BA [74]	Inertia factor	[0, 1]

### 4.3. Feature Extraction Evaluation

The evaluation of the feature extraction was performed, and the results are presented and discussed in this section. Table 4 presents the evaluation results of the feature extraction process. The feature extraction is performed in terms of four deep learning models, and the classification results are recorded in the table. This table shows that the results achieved by the GoogleNet deep model are superior to the other deep learning models. Therefore, the GoogleNet deep model is adopted for further steps in the proposed methodology.

### 4.4. Feature Selection Evaluation

Once the features are extracted, the best set of features that can be used to classify the input image are selected. The feature selection is performed using the proposed algorithm presented in the previous section. Eleven other feature selection algorithms are employed for comparison to prove the effectiveness and superiority of the proposed feature selection method. The evaluation of the results achieved by the proposed and other feature selection methods is presented in Table 5. This table shows that the results achieved by the proposed feature selection method are much better than those of the other methods.

In addition, a statistical analysis is applied to study the stability of the proposed feature selection methods, and the results are listed in Table 6. In this table, the proposed feature selection methods achieved the minimum standard deviation of the best mean, median, minimum, and maximum measurements compared to the other feature selection methods.

On the other hand, two statistical tests were conducted to study the statistical difference between the proposed feature selection method and the other feature selection methods. The first test is the one-way analysis of variance (ANOVA) test, and the other test is the Wilcoxon signed-rank test. The results of these tests are presented in Table 7 and Table 8. As presented in these tables, the most significant parameter is p, which indicates a statistical difference when its value is less than 0.005. The results presented in these tables confirm the statistical difference between the proposed feature selection method and the other feature selection methods.

**Figure 5:**
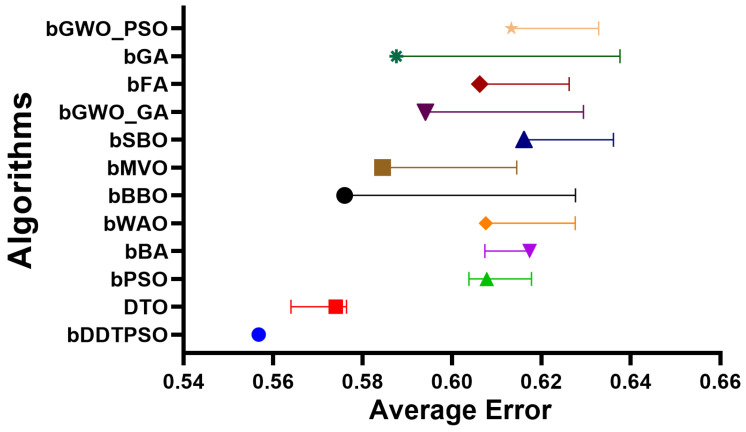
The average error achieved by the proposed feature selection method compared to other methods.

**Figure 6:**
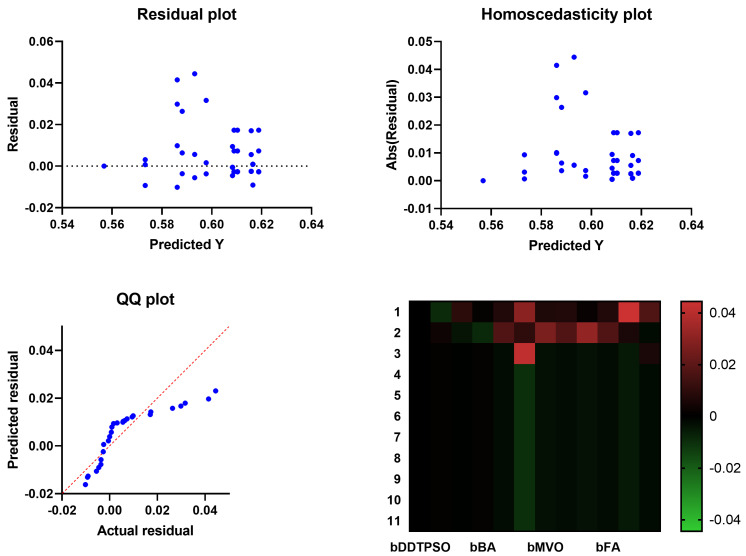
Analysis plots of the results achieved by the proposed feature selection algorithm bDDTPSO.

A more in-depth analysis of the results achieved by the proposed feature selection method is presented in terms of the plots shown in Figure 5 and Figure 6. These plots represent the average error the proposed feature selection method achieves compared to the other feature selection methods. As shown in this plot, the proposed method is superior and more robust in selecting the best set of features that can be used to classify breast cancer cases robustly. More analysis of the robustness of the proposed approach is represented by the residual, homoscedasticity, quartile–quartile (QQ), and heatmap plots of Figure 6. In the residual and homoscedasticity plots, the error values are in the range from 0.01 to 0.04, indicating the proposed approach’s accuracy. In addition, the results of the QQ plot are aligned close to the red diagonal line, confirming the accuracy of the proposed method. On the other hand, a set of 11 runs are performed to study the average error of the proposed method with comparison to the other methods. In the heatmap (bottom-right) plot, the results achieved by the proposed method are superior to the results of the other methods. These plots’ results emphasize the proposed method’s effectiveness and superiority.

### 4.5. Evaluation of Classification Models

The best set of features selected by the proposed feature selection algorithm are then used to train the classification models. In this work, four classifiers are evaluated to choose the best classifier. These classifiers are convolutional neural networks (CNN), neural networks (NN), support vector machines (SVM), and K-nearest neighbors (KNN). The results of these classifiers are presented in Table 9. In this table, the performance of the CNN model is much better than the other models is and thus adopted for optimization to further improve its performance.

### 4.6. Evaluation of Optimized CNN Classification

As the CNN model is adopted for the classification of the input images, this model is subjected to optimization using the proposed DDTPSO algorithm to improve its performance. The optimization of this model is performed using four optimization algorithms in addition to the proposed optimization algorithm. The results of the optimized CNN using these optimization algorithms are presented in Table 10. This table shows that the performance of the optimized CNN is much better than the CNN without optimization, especially using the proposed DDTPSO optimization algorithm. These results confirm the effectiveness of the proposed optimization algorithm.

To further analyze the proposed optimization algorithm’s performance, the optimized CNN’s results using different optimization methods are analyzed statistically to show the proposed algorithm’s superiority and statistical difference. Table 11 presents the results of the statistical analysis, which confirm the stability of the optimization of CNN using the proposed algorithm with the minimum value of standard deviation and the best values achieved for the other criteria in the statistical analysis.

The statistical difference of the proposed optimization algorithm is studied in terms of the ANOVA and Wilcoxon tests, similar to the study performed on the proposed feature selection method discussed in the previous section. The results of these tests are presented in Table 12 and Table 13. The p values in these tables are less than 0.005, proving the statistical difference between the proposed optimization algorithm and the other algorithms.

**Figure 7:**
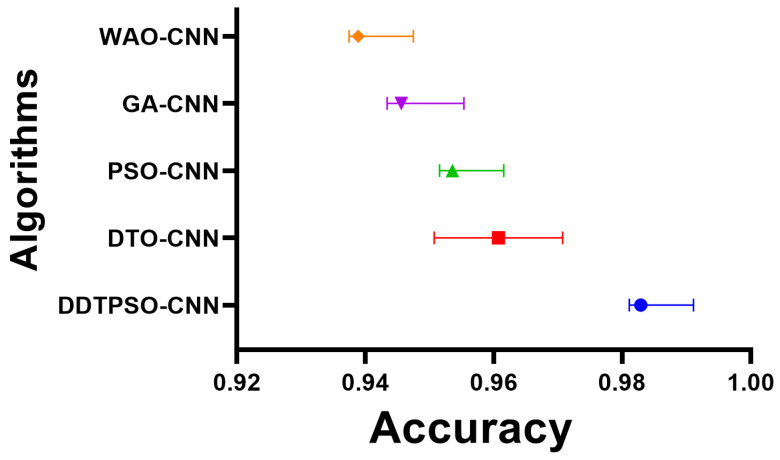
Accuracy of the results achieved by CNN optimization using the proposed DDTPSO method compared to other methods.

**Figure 8:**
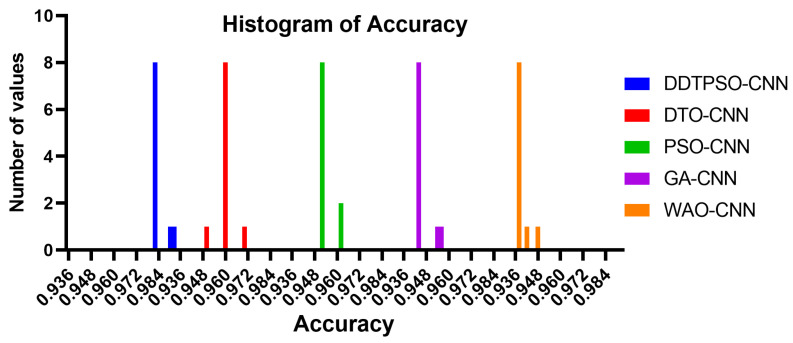
Histogram of the accuracy achieved by the proposed method compared to other methods.

**Figure 9:**
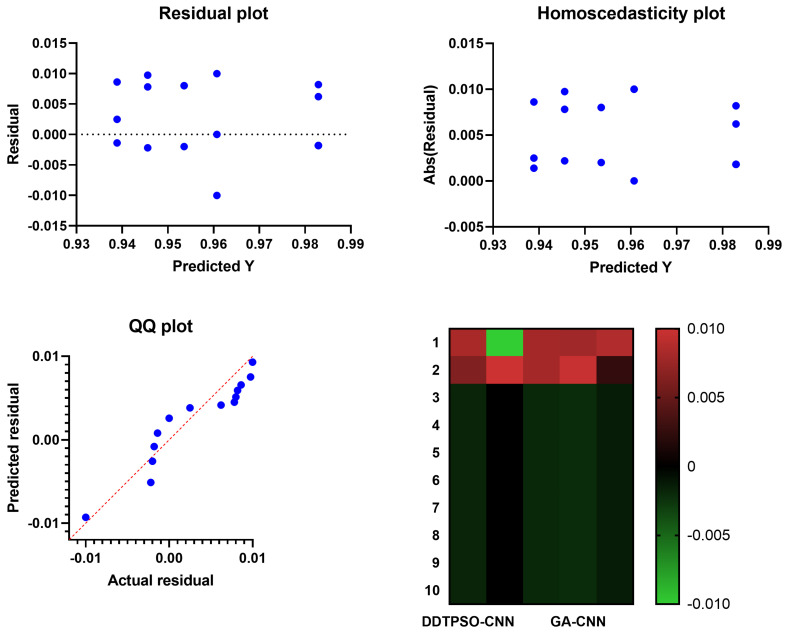
Analysis plots of the results achieved by the proposed DDTPSO optimization algorithm when applied to CNN.

Another in-depth analysis of the results achieved by the proposed optimization method for CNN is presented in terms of the plots shown in Figure 7, Figure 8 and Figure 9. These plots represent the accuracy, histogram of accuracy, and analysis plots achieved by the proposed optimization of CNN compared to the other optimization methods. These plots show that the proposed method is superior and more robust in classifying breast cancer cases.

**Figure 10:**
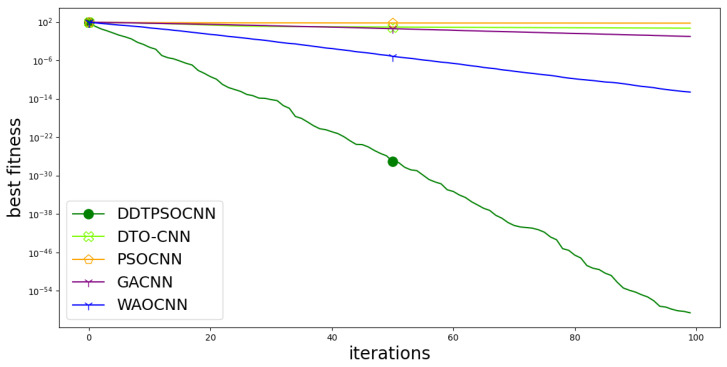
Convergence curve of the proposed method with comparison to other methods.

On the other hand, the convergence curve is depicted in Figure 10. This curve is used to study the cost function values versus the computation time. In the figure, it can be noted that the proposed approach is converging faster than the other competitor methods. This result gives additional emphasize of the superiority of the proposed methodology.

In addition, the linear regression test is performed to study the relationship between the proposed and other approaches. The regression test results are presented in Table 14. In this table, the calculated *p* value is less than 0.05, indicating the significant relationship between the proposed methodology and the competitor methodologies.

## 5. Conclusions

This paper proposes a fully automated technique to analyze ultrasound images for signs of breast cancer. The proposed approach relies on a series of consecutive procedures. At first, a GoogleNet deep learning model is applied to the breast ultrasound data, enhancing and retraining it. Therefore, a novel optimization technique is derived from the dipper throated optimization algorithm and the particle swarm optimization algorithm to select the best set of features. This set of features is used in classifying breast cancer cases. Experimental results showed that the proposed approach achieved the highest accuracy, at 98.1% (using the selected features and CNN classifier). Compared to more modern methods, the outcomes are better when the proposed framework is used. This work’s strengths lie in the enhanced dataset, thereby increasing the training strength. In addition, the irrelevant features were eliminated through the selection of best features using a novel feature selection algorithm. In the future, two main steps can be considered: utilizing a dataset of larger size and (ii) developing a new CNN model specifically for breast cancer classification.

## Figures and Tables

**Table 1:** A review of classification methods for breast cancer.

Article	Approach	Dataset	Features
[56]	Fuzzy logic and U-Net	BUSI	CNN features
[14]	deep representation scaling and CNN	BUSI	Deep features through scaling layers
[49]	Radiomics and Machine learning	BUSI	Geometric features and Textural
[3]	Semantic segmentation and Fuzzy logic	BUSI	Deep features
[50]	U-Net Encoder-Decoder CNN	BUSI	High level contextual features
[48]	Watershed and Hilbert transform	BUSI	Textural features
[47]	Shape Adaptive CNN	Breast Ultrasound Images	Deep features

**Table 3:** Evaluation metrics used in assessing the proposed methods.

Metric	Value
Mean	$\frac{1}{M}\sum_{i=1}^{M}S_{i}^{*}$
Best fitness	$min_{i=1}^{M}S_{i}^{*}$
Worst fitness	$max_{i=1}^{M}S_{i}^{*}$
Average fitness size	$\frac{1}{M}\sum_{i=1}^{M}size\left(S_{i}^{*}\right)$
Average error	$\frac{1}{M}\sum_{j=1}^{M}\frac{1}{N}\sum_{i=1}^{N}mse\left(\hat{V_{i}}-V_{i}\right)$
Standard deviation	$\sqrt{\frac{1}{M-1}\sum_{i=1}^{M}{\left(S_{i}^{*}-Mean\right)}^{2}}$
Accuracy	$\frac{TP+TN}{TP+TN+FP+FN}$
*p* value	$\frac{TP}{TP+FP}$
N value	$\frac{TN}{TN+FN}$
Specificity	$\frac{TN}{TN+FP}$
Sensitivity	$\frac{TP}{TP+FN}$
F Score	$\frac{TP}{TP+0.5(FP+FN)}$

**Table 4:** Evaluation of the results achieved by various deep learning models.

	Accuracy	Sensitivity	Specificity	*p* Value	N Value	F Score
VGG16Net	0.776	0.750	0.778	0.231	0.972	0.353
ResNet-50	0.789	0.769	0.791	0.250	0.974	0.377
AlexNet	0.828	0.811	0.830	0.273	0.982	0.408
GoogLeNet	0.853	0.784	0.857	0.266	0.984	0.397

**Table 5:** Evaluation of the resutls achieved by the feature selection methods.

	bDDTPSO	DTO	bPSO	bBA	bWAO	bBBO	bMVO	bSBO	bFA	bGA	bGWO_PSO	bGWO_GA
Average error	0.557	0.574	0.608	0.617	0.608	0.576	0.584	0.616	0.606	0.588	0.613	0.594
Average select size	0.510	0.710	0.710	0.849	0.873	0.873	0.806	0.880	0.744	0.652	0.843	0.632
Average fitness	0.620	0.636	0.635	0.657	0.642	0.640	0.664	0.674	0.686	0.648	0.644	0.642
Best fitness	0.522	0.556	0.615	0.547	0.606	0.630	0.589	0.617	0.605	0.551	0.598	0.620
Worst fitness	0.620	0.623	0.683	0.649	0.683	0.716	0.707	0.697	0.703	0.666	0.708	0.696
Std. fitness	0.442	0.447	0.446	0.456	0.449	0.491	0.497	0.507	0.483	0.449	0.465	0.448

**Table 6:** Statistical analysis of the results achieved by the feature selection methods.

	bDDTPSO	DTO	bPSO	bBA	bWAO	bBBO	bMVO	bSBO	bFA	bGA	bGWO_PSO	bGWO_GA
Number of values	11	11	11	11	11	11	11	11	11	11	11	11
75% Percentile	0.557	0.574	0.608	0.617	0.608	0.596	0.585	0.616	0.606	0.588	0.613	0.594
25% Percentile	0.557	0.574	0.608	0.617	0.608	0.576	0.585	0.616	0.606	0.588	0.613	0.594
Maximum	0.557	0.576	0.618	0.617	0.628	0.628	0.615	0.636	0.626	0.638	0.633	0.629
Median	0.557	0.574	0.608	0.617	0.608	0.576	0.585	0.616	0.606	0.588	0.613	0.594
Minimum	0.557	0.564	0.604	0.607	0.608	0.576	0.585	0.616	0.606	0.588	0.613	0.594
Range	0.000	0.012	0.014	0.010	0.020	0.052	0.030	0.020	0.020	0.050	0.020	0.035
Std. error of mean	0.000	0.001	0.001	0.001	0.002	0.006	0.003	0.002	0.002	0.005	0.002	0.003
Std. deviation	0.000	0.003	0.003	0.003	0.006	0.019	0.009	0.006	0.006	0.015	0.006	0.011
Mean	0.557	0.573	0.608	0.617	0.610	0.586	0.588	0.619	0.609	0.593	0.616	0.598

**Table 7:** One-way analysis of variance (ANOVA) test of the feature selection methods compared to the proposed feature selection method.

	SS	DF	MS	F (DFn, DFd)	*p* Value
Treatment	0.04443	11	0.004039	F (11, 120) = 49.80	*p* < 0.0001
Residual	0.009733	120	0.00008111		
Total	0.05416	131			

**Table 8:** Wilcoxon signed-rank test of the feature selection methods compared to the proposed feature selection method.

	bDDTPSO	DTO	bPSO	bBA	bWAO	bBBO	bMVO	bSBO	bFA	bGA	bGWO_PSO	bGWO_GA
Theoretical median	0	0	0	0	0	0	0	0	0	0	0	0
Actual median	0.5568	0.574	0.6078	0.6174	0.6076	0.576	0.5845	0.6161	0.6062	0.5876	0.6133	0.5941
Sum of negative ranks	0	0	0	0	0	0	0	0	0	0	0	0
Sum of signed ranks (W)	66	66	66	66	66	66	66	66	66	66	66	66
Sum of positive ranks	66	66	66	66	66	66	66	66	66	66	66	66
Significant (alpha = 0.05)?	Yes	Yes	Yes	Yes	Yes	Yes	Yes	Yes	Yes	Yes	Yes	Yes
Discrepancy	0.5568	0.574	0.6078	0.6174	0.6076	0.576	0.5845	0.6161	0.6062	0.5876	0.6133	0.5941
*p* value (two tailed)	0.001	0.001	0.001	0.001	0.001	0.001	0.001	0.001	0.001	0.001	0.001	0.001
Number of values	11	11	11	11	11	11	11	11	11	11	11	11

**Table 9:** Evaluation of the results achieved by various classification models.

	Accuracy	Sensitivity	Specificity	*p* Value	N Value	F Score
CNN	0.925	0.300	0.981	0.588	0.940	0.397
NN	0.917	0.300	0.972	0.492	0.940	0.373
SVM	0.894	0.333	0.947	0.375	0.938	0.353
KNN	0.884	0.727	0.897	0.364	0.976	0.485

**Table 10:** Evaluation results of the optimized CNN for classifying breast cancer cases.

	Accuracy	Sensitivity	Specificity	*p* Value	N Value	F Score
DDTPSO-CNN	0.981	0.984	0.981	0.865	0.998	0.920
DTO-CNN	0.961	0.982	0.949	0.914	0.990	0.947
PSO-CNN	0.952	0.982	0.927	0.914	0.985	0.947
GA-CNN	0.943	0.980	0.909	0.909	0.980	0.943
WAO-CNN	0.938	0.980	0.889	0.909	0.976	0.943

**Table 11:** Statistical analysis of the results achieved by the optimized CNN.

	DDTPSO-CNN	DTO-CNN	PSO-CNN	GA-CNN	WAO-CNN
Number of values	10	10	10	10	10
Minimum	0.981	0.951	0.952	0.943	0.938
Mean	0.983	0.961	0.954	0.946	0.939
Median	0.981	0.961	0.952	0.943	0.938
Maximum	0.991	0.971	0.962	0.955	0.948
75% Percentile	0.983	0.961	0.954	0.946	0.939
25% Percentile	0.981	0.961	0.952	0.943	0.938
Std. error of mean	0.0012	0.0015	0.0013	0.0015	0.0010
Std. deviation	0.0038	0.0047	0.0042	0.0046	0.0033
Range	0.010	0.020	0.010	0.012	0.010

**Table 12:** Wilcoxon signed-rank test of the optimization models compared to the proposed optimization model.

	DDTPSO-CNN	DTO-CNN	PSO-CNN	GA-CNN	WAO-CNN
Theoretical median	0	0	0	0	0
Actual median	0.9811	0.9607	0.9516	0.9434	0.9375
Sum of negative ranks	0	0	0	0	0
Sum of signed ranks (W)	55	55	55	55	55
Sum of positive ranks	55	55	55	55	55
*p* value (two tailed)	0.002	0.002	0.002	0.002	0.002
Exact or estimate	Exact	Exact	Exact	Exact	Exact
Discrepancy	0.9811	0.9607	0.9516	0.9434	0.9375
Significant (alpha = 0.05)?	Yes	Yes	Yes	Yes	Yes
Number of values	10	10	10	10	10

**Table 13:** One-way analysis of variance (ANOVA) test of the optimization models compared to the proposed optimization model.

	SS	DF	MS	F (DFn, DFd)	*p* Value
Treatment	0.01153	4	0.002882	F (4, 45) = 165.9	*p* < 0.0001
Residual	0.0007819	45	0.00001738		
Total	0.01231	49			

**Table 14:** Linear regression test results.

Linear Regression	DTO-CNN	PSO-CNN	GA-CNN	WAO-CNN
Best-fit values				
Slope	−0.1515	1.094	1.186	0.8053
Y-intercept	1.11	−0.1217	−0.2197	0.1473
X-intercept	7.326	0.1113	0.1853	−0.1829
1/slope	−6.603	0.9141	0.8435	1.242
Std. error				
Slope	0.4324	0.04789	0.09457	0.09921
Y-intercept	0.425	0.04707	0.09295	0.09751
95% confidence intervals				
Slope	−1.149 to 0.8457	0.9835 to 1.204	0.9675 to 1.404	0.5766 to 1.034
Y-intercept	0.1294 to 2.090	−0.2303 to −0.01317	−0.4341 to −0.005363	−0.07754 to 0.3722
X-intercept	1.819 to +infinity	0.01339 to 0.1912	0.005543 to 0.3092	−0.6455 to 0.07498
Goodness of fit				
R square	0.0151	0.9849	0.9516	0.8917
Sy.x	0.004962	0.0005496	0.001085	0.001138
Is slope significantly non-zero?				
F	0.1227	521.8	157.2	65.9
DFn, DFd	1, 8	1, 8	1, 8	1, 8
*p* value	<0.0001	<0.0001	<0.0001	<0.0001
Deviation from zero	Significant	Significant	Significant	Significant

## Data Availability

Not applicable.
